# ﻿Three new species of *Neopestalotiopsis* and *Pseudopestalotiopsis* (Sporocadaceae, Amphisphaeriales) associated with shrub leaf diseases from Fujian, China

**DOI:** 10.3897/mycokeys.119.148647

**Published:** 2025-06-24

**Authors:** Zhi-Ang Heng, Tai-Chang Mu, Nemat O. Keyhani, Li-Xia Yang, Ming-Hai Zheng, Hua-Jun Lv, Zhi-Ying Zhao, Yu-Chen Mao, Jun-Ya Shang, Jiao Yang, Hui-Li Pu, Yong-Sheng Lin, Meng-Jia Zhu, Yu-Xiao Dang, Dong-Mei Wu, Zhen-Xing Qiu, Jun-Zhi Qiu, Xia-Yu Guan

**Affiliations:** 1 State Key Laboratory of Agricultural and Forestry Biosecurity, College of Life Sciences, Fujian Agriculture and Forestry University, Fuzhou 350002, China; 2 Department of Biological Sciences, University of Illinois, Chicago 60607, USA; 3 Biotechnology Research Institute, Xinjiang Academy of Agricultural and Reclamation Sciences, Shihezi 832061, China; 4 College of Literature and Law, Fuzhou Technology and Business University, Fuzhou 350715, China; 5 Key Laboratory of Ministry of Education for Genetics, Breeding and Multiple Utilization of Crops, College of Horticulture, Fujian Agriculture and Forestry University, Fuzhou 350002, China

**Keywords:** Morphology, multigene phylogeny, new taxa, pestalotioid fungi

## Abstract

*Neopestalotiopsis* and *Pseudopestalotiopsis* are classified as pestalotioid fungi, include a diverse range of plant pathogenic, endophytic and saprobic species and are widely distributed in tropical and temperate climates. These fungi are associated with a wide variety of plants worldwide and are exemplified by multi-septate conidia with appendages at both ends. Phytopathogenic members cause various plant diseases, for example, leaf spot, fruit rot, canker, blight and various infections affecting palm, mango, blueberry, tea and other important crops. In this study, six isolates were collected from diseased leaves of *Litseaverticillata*, *Ixorachinensis* and an unidentified shrub in Fujian Province, China. Based on morphological characteristics and molecular phylogenetic analyses of combined nucleotide sequences of internal transcribed spacer regions of rDNA (ITS), the partial translation elongation factor 1-alpha gene (*tef1*) and partial beta-tubulin gene (*tub2*), three new species *Neopestalotiopsislitseae***sp. nov.**, *Neo.longqishanensis***sp. nov.** and *Pseudopestalotiopsiszhangzhouensis***sp. nov.** are described and illustrated herein.

## ﻿Introduction

Pestalotioid fungi represent a diverse group of Ascomycota, common in tropical and temperate regions, that often associate with plants as pathogens, endophytes and/or saprophytes (Guba 1961; Barr 1975; Nag Raj 1993; Maharachchikumbura et al. 2014a). Pathogenic pestalotioid fungi that cause plant diseases result in symptoms that can include cankers, leaf spots, shoot and stem dieback and fruit rots. In rare cases, these fungi have also been implicated in human diseases, for example, onychomycosis (Borgohain et al. 2020). Species identification of pestalotioid fungi remains a major challenge due to taxonomic confusion and homonyms as a result of significant morphological overlap between species (Jeewon et al. 2002, 2003; Maharachchikumbura et al. 2011, 2012, 2014b). The genus *Pestalotia* was originally characterised by fusiform conidia with six-celled structures and appendages at both apical and basal ends (De Notaris 1839). A century later, Steyaert (1955) reclassified the genus into three distinct genera — *Pestalotia*, *Pestalotiopsis*, and *Truncatella* — based on conidial cell counts. This taxonomic framework remains widely adopted (Maharachchikumbura et al. 2011), though debates regarding generic boundaries within pestalotioid fungi have occurred (Maharachchikumbura et al. 2014b). Prior to the 1990s, the taxonomy of pestalotioid fungi relied on stable conidial traits, particularly the pigmentation of the three median cells — versicolorous in *Neopestalotiopsis* and concolorous in other genera (Maharachchikumbura et al. 2011, 2012). However, the use of conidial morphology for species identification can be contentious due to high variability in colony features (colour, texture, shape) and conidial characteristics under differing culture conditions (Egger 1995; Hu et al. 2007). In particular, significant overlap of conidial features makes it difficult to identify pestalotioid fungal species solely based on morphology. Recent taxonomic revisions, incorporating 18S rRNA gene sequencing and multi-loci analyses (Maharachchikumbura et al. 2014b), have helped to resolve three distinct lineages and has led to the establishment of two new genera, *Neopestalotiopsis* and *Pseudopestalotiopsis* (Maharachchikumbura et al. 2014b; Tsai et al. 2018). Thus, the combination of morphological characterisation and molecular sequence data (e.g. multigene phylogenies) have become the standard for accurate identification within the pestalotioid group and for delineating members within these three genera (Norphanphoun et al. 2019).

Specifically, *Neopestalotiopsis* and *Pseudopestalotiopsis* were separated from *Pestalotiopsis* due to differences in their conidia and ITS length (and sequence). Morphologically, *Neopestalotiopsis* is distinguished from *Pestalotiopsis* and *Pseudopestalotiopsis* by the versicolourous intermediate cells (Maharachchikumbura et al. 2014b) and *Pseudopestalotiopsis* differs from *Pestalotiopsis* by having darker three intermediate cells and knobbed apical appendage (Maharachchikumbura et al. 2014b). In recent years, several new species, for example, *Pestalotiopsiscamelliae*, *Pseudopestalotiopsisignota* and *Ps.theae*, have been introduced into the group (Maharachchikumbura et al. 2016; Liu et al. 2017; Nozawa et al. 2017; Tibpromma et al. 2018; Tsai et al. 2018; Watanabe et al. 2018).

*Litseaverticillata* (Lauraceae) is an evergreen shrub or small tree and is often harvested from the wild for local use as a medicine and/or source of fuel (Yan et al. 2024) and *Ixorachinensis* (Rubiaceae) is a salt-tolerant shrub that primarily is found in wet tropical regions, producing pink and white blossoms attractive to butterflies and hummingbirds. Here, we report on the taxonomic assignment and identification of fungi found on diseased leaves of these plants, based on a combination of morphological and molecular phylogenetic analyses, the latter of which included analysis of multi-locus nucleotide sequencing data, examining the internal transcribed spacer region of rDNA (ITS), the translation elongation factor 1-alpha gene (*tef1*) and the beta-tubulin gene (*tub2*) genetic loci. In total, we have identified two new species of *Neopestalotiopsis*, one isolated from *Litseaverticillata* and the other from an unknown plant and one new species of *Pseudopestalotiopsis* isolated from *Ixorachinensis*, all found in Fujian Province, China.

## ﻿Materials and methods

### ﻿Sample collection, isolation and morphological observations

Diseased leaves derived from *L.verticillata*, *I.chinensis* and an unknown shrub were collected from Sanming and Zhangzhou City of Fujian Province, China, in September 2023. The leaf samples were processed as described previously (Fu et al. 2019). Tissue fragments (~ 25 mm^2^) were taken from the margin of leaves at sites of apparent infection by fungi. Samples were surface disinfected by immersion in 75% ethanol solution for 60 s, placed in sterile deionised water for 45 s, transferred to 5% sodium hypochlorite solution for 30 s and then rinsed three times in sterile deionised water for 60 s. The leaf fragments were then dried with sterilised filter paper and transferred on to potato dextrose agar (PDA) media plates (deionised water 1,000 ml, potato 200 g, agar 20 g, dextrose 20 g, pH ~7.0, available after sterilisation) and incubated at 25 °C for 5–7 d. Single colonies of the isolated fungi were purified by repeated streaking on PDA grown as above. Samples of the dried specimens were deposited in the Herbarium Mycologicum Academiae Sinicae, Institute of Microbiology, Chinese Academy of Sciences, Beijing, China (HMAS). Living cultures were conserved in the China General Microbiological Culture Collection Center (CGMCC). Images of colony morphologies were captured using a digital camera (Canon EOS 6D Mark II, Tokyo, Japan) at 7 and 14 d after inoculation on indicated media plates (Cai et al. 2009). Fungal micromorphological features were observed and photographed using a stereomicroscope (Nikon SMZ745, Tokyo, Japan) and a biomicroscope (Ni-U, Tokyo, Japan) coupled to digital cameras (Olympus, Tokyo, Japan). Image analyses were performed using the Digimizer 5.4.4 software (https://www.digimizer.com). All fungal strains were stored in 10% sterilised glycerine and sterile water at 4 °C in 2.0 ml tubes. Taxonomic information of the new taxa was registered in MycoBank (http://www.mycobank.org, accessed on 15 November 2024).

### ﻿DNA extraction, PCR amplification and sequencing

Fungal genomic DNA was extracted from growing mycelium using the Fungal DNA Mini Kit (OMEGA-D3390, Feiyang Biological Engineering Co., Ltd., Guangzhou, China) according to the manufacturer’s instructions. Indicated genetic loci were amplified and isolated by polymerase chain reaction (PCR). Target genetic loci included regions of rDNA (ITS), beta-tubulin (*tub2*) and translation elongation factor-alpha (*tef1*) genes. The PCR thermal cycling program and primer pairs are given in Table 1. PCR reaction volumes were 25 µl containing: 12.5 µl of 2 × Rapid Taq Master Mix (Vazyme Nanjing, China), 1 µl (10 µM) each of forward and reverse primers (Sangon, Shanghai, China), 1 µl of template genomic DNA and 9.5 µl of double-distilled water (ddH_2_O) using Bio-Rad Thermocycler (Hercules, CA, USA) for amplification. The integrity and size of all PCR products were checked on 1% agarose gel electrophoresis and the products were sequenced by a commercial company (Tsingke Co., Ltd, Fuzhou, China). The forward and reverse sequences of PCR products for each locus were processed by MEGA 7.0.20 software (Kumar et al. 2016). The new sequences generated in this study have been deposited in GenBank (https://www.ncbi.nlm.nih.gov, Table 2).

**Table 1.** Target loci, primers and PCR thermal cycle programmes.

Locus	Primers	Sequence (5′-3′)	PCR Cycles
ITS	ITS5 ITS4	GGAAGTAAAAGTCGTAACAAGG TCCTCCGCTTATTGATATGC	94 °C: 3 min, (94 °C: 15 s, 54 °C: 15 s, 72 °C: 30 s) × 35 cycles, 72 °C: 5 min
*tub2*	T1 Bt2b	AACATGCGTGAGATTGTAAGT ACCCTCAGTGTAGTGACCCTTGGGC	95 °C: 3 min, (94 °C: 30 s, 55 °C: 50 s, 72 °C: 1 min) × 35 cycles, 72 °C: 7 min
*tef1*	EF1-728F	CATCGAGAAGTTCGAGAAGG GGARGTACCAGTSATCATGTT	94 °C: 5 min, (94 °C: 30 s, 52 °C: 30 s, 72 °C: 30 s) × 35 cycles, 72 °C: 7 min
	EF2		

**Table 2.** Species names, strain number, substrate or host, locations and corresponding GenBank accession numbers of DNA sequences used in the molecular phylogenetic analyses of *Neopestalotiopsis*.

Species	Specimen voucher /Strain	Host/Substrate	Locations	GenBank Accession Number			References
				ITS	*tub2*	*tef1*	
*Neopestalotiopsisacrostichi*	MFLUCC 17-1754^T^	*Acrostichumaureum*	Thailand	MK764272	MK764338	MK764316	Norphanphounet et al. (2019)
*Neo.acrostichi*	MFLUCC 17-1755	*Acrostichumaureum*	Thailand	MK764273	MK764339	MK764317	Norphanphounet et al. (2019)
*Neo.ageratinae*	CGMCC 3.23468 = LC11319^T^	*Ageratinaadenophora*	China	OR247899	OR381006	OR361406	Razaghi et al. (2024)
*Neo.ageratinae*	LC15845	*Ageratinaadenophora*	China	OR247896	OR381007	OR361407	Razaghi et al. (2024)
*Neo.alpapicalis*	MFLUCC 17-2544^T^	*Rhizophoramucronata*	Thailand	MK357772	MK463545	MK463547	Kumar et al. (2019)
*Neo.alpapicalis*	MFLUCC 17-2545	*Rhizophoramucronata*	Thailand	MK357773	MK463546	MK463548	Kumar et al. (2019)
*Neo.amomi*	HKAS 124563^T^	*Amomumvillosum*	China	OP498012	OP752133	OP653489	Sun et al. (2023)
*Neo.amomi*	HKAS 124564	*Amomumvillosum*	China	OP498013	OP765913	OP753382	Sun et al. (2023)
*Neo.aotearoa*	CBS 367.54^T^	Canvas	New Zealand	KM199369	KM199454	KM199526	Maharachchikumbura et al. (2014b)
*Neo.asiatica*	MFLUCC 12-0286^T^	Unidentified tree	China	JX398983	JX399018	JX399049	Maharachchikumbura et al. (2012)
*Neo.australis*	CBS 114159^T^	*Telopea* sp.	Australia	KM199348	KM199432	KM199537	Maharachchikumbura et al. (2014b)
*Neo.brachiata*	MFLUCC 17-1555^T^	*Rhizophoraapiculata*	Thailand	MK764274	MK764340	MK764318	Norphanphoun et al. (2019)
*Neo.brasiliensis*	COAD 2166^T^	*Psidiumguajava*	Brazil	MG686469	MG692400	MG692402	Bezerra et al. (2018)
*Neo.camelliae-oleiferae*	CSUFTCC81^T^	*Camelliaoleifera*	China	OK493585	OK562360	OK507955	Li et al. (2021)
*Neo.camelliae-oleiferae*	CSUFTCC82	*Camelliaoleifera*	China	OK493586	OK562361	OK507956	Li et al. (2021)
*Neo.castanopsidis*	CGMCC 3.23478 = LC13333^T^	*Castanopsisboisii*	China	OR247897	OR381018	OR361418	Razaghi et al. (2024)
*Neo.castanopsidis*	LC15849	*Castanopsisboisii*	China	OR247895	OR381019	OR361419	Razaghi et al. (2024)
*Neo.cavernicola*	KUMCC 20-0269^T^	Cave	China	MW545802	MW557596	MW550735	Liu et al. (2021)
*Neo.cavernicola*	KUMCC 20-0332	Cave	China	MW581238	MW590328	MW590327	Liu et al. (2021)
*Neo.celtidis*	CGMCC 3.23513 = LC8947^T^	*Celtissinensis*	China	OR247900	OR381049	OR361449	Razaghi et al. (2024)
*Neo.celtidis*	LC15870	*Celtissinensis*	China	OR247894	OR381050	OR361450	Razaghi et al. (2024)
*Neo.chiangmaiensis*	MFLUCC 18-0113^T^	*Pandanus* sp.	Thailand	*_*	MH412725	MH388404	Tibpromma et al. (2018)
*Neo.chrysea*	MFLUCC 12-0261^T^	Dead leaves	China	JX398985	JX399020	JX399051	Maharachchikumbura et al. (2012)
*Neo.chrysea*	MFLUCC 12-0262	Dead plant	China	JX398986	JX399021	JX399052	Maharachchikumbura et al. (2012)
*Neo.clavispora*	MFLUCC 12-0281^T^	*Magnolia* sp.	China	JX398979	JX399014	JX399045	Maharachchikumbura et al. (2012)
*Neo.clavispora*	MFLUCC 12-0280	*Magnolia* sp.	China	JX398978	JX399013	JX399044	Maharachchikumbura et al. (2012)
*Neo.clavispora*	CBS 447.73	Decaying wood	Sri Lanka	KM199374	KM199443	KM199539	Maharachchikumbura et al. (2014b)
*Neo.cocoës*	MFLUCC 15-0152^T^	*Cocosnucifera*	Thailand	KX789687	*_*	KX789689	Hyde et al. (2016)
*Neo.coffeae-arabicae*	HGUP4019^T^	*Coffeaarabica*	China	KF412649	KF412643	KF412646	Song et al. (2013)
*Neo.coffeae-arabicae*	HGUP4015	*Coffeaarabica*	China	KF412647	KF412641	KF412644	Song et al. (2013)
*Neo.collariata*	CGMCC 3.23493 = LC4212^T^	*Rhododendron* sp.	China	OR247905	OR381026	OR361426	Razaghi et al. (2024)
*Neo.collariata*	LC4276	*Rhododendron* sp.	China	OR247904	OR381028	OR361428	Razaghi et al. (2024)
*Neo.collariata*	LC4205	*Rhododendron* sp.	China	OR247906	OR381022	OR361422	Razaghi et al. (2024)
*Neo.collariata*	LC8308	*Diospyroskaki*	China	OR247902	OR381044	OR361444	Razaghi et al. (2024)
*Neo.concentrica*	CFCC 55162^T^	*Rosarugosa*	China	OK560707	OM117698	OM622433	Peng et al. (2022)
*Neo.concentrica*	CFCC 55163	*Rosachinensis*	China	OK560708	OM117699	OM622434	Peng et al. (2022)
*Neo.cubana*	CBS 600.96^T^	Leaf litter	China	KM199347	KM199438	KM199521	Maharachchikumbura et al. (2014b)
*Neo.dendrobii*	MFLUCC 14-0106^T^	*Dendrobiumcariniferum*	Thailand	MK993571	MK975835	MK975829	Ma et al. (2019)
*Neo.dendrobii*	MFLUCC 14-0132	*Dendrobium* sp.	Thailand	MK993572	*_*	MK975830	Ma et al. (2019)
*Neo.dendrobii*	MFLUCC 14-0099	*Dendrobiumcariniferum*	Thailand	MK993570	MK975834	MK975828	Ma et al. (2019)
*Neo.dimorphospora*	CGMCC 3.23497 = LC4444^T^	*Euryachinensis*	China	OR247903	OR381030	OR361430	Razaghi et al. (2024)
*Neo.dimorphospora*	LC8359	*Patriniavillosa*	China	OR247901	OR381045	OR361445	Razaghi et al. (2024)
*Neo.dolichoconidiophora*	CGMCC 3.23490 = LC3634^T^	*Cycasrevoluta*	China	OR247911	OR381021	OR361421	Razaghi et al. (2024)
*Neo.dolichoconidiophora*	LC12283	Aucubajaponicavar.variegata	China	OR247898	OR381008	OR361408	Razaghi et al. (2024)
*Neo.drenthii*	BRIP 72264a^T^	*Macadamiaintegrifolia*	Australia	MZ303787	MZ312680	MZ344172	Prasannath et al. (2021)
*Neo.drenthii*	BRIP 72263a	*Macadamiaintegrifolia*	Australia	MZ303786	MZ312679	MZ344171	Prasannath et al. (2021)
*Neo.egyptiaca*	CBS 140162^T^	*Mangiferaindica*	Egypt	KP943747	KP943746	KP943748	Crous et al. (2015)
*Neo.egyptiaca*	COAD 2167	*Psidiumguajava*	Brazil	MG686470	MG692401	MG692403	Bezerra et al. (2018)
*Neo.elaeagni*	HGUP10002^T^	*Elaeagnuspungens*	China	MW930716	MZ683391	MZ203452	He et al. (2022)
*Neo.elaeagni*	HGUP10006	*Elaeagnuspungens*	China	ON597079	ON595537	ON595535	He et al. (2022)
*Neo.elaeidis*	MFLUCC 15-0735^T^	*Elaeisguineensis*	Thailand	ON650690	*_*	ON734012	Konta et al. (2023)
*Neo.elaeidis*	MFLUCC 15-0801	*Elaeisguineensis*	Thailand	ON650689	*_*	ON734011	Konta et al. (2023)
*Neo.ellipsospora*	MFLUCC 12-0283^T^	Dead plant materials	China	JX398980	JX399016	JX399047	Maharachchikumbura et al. (2012)
*Neo.ellipsospora*	MFLUCC 12-0284	Dead plant materials	China	JX398981	JX399015	JX399046	Maharachchikumbura et al. (2012)
*Neo.ellipsospora*	CBS 115113	*Ardisiacrenata*	China	KM199343	KM199450	KM199544	Maharachchikumbura et al. (2014b)
*Neo.eucalypti*	PA3	*Eucalyptus* sp.	Brazil	*_*	MK286942	MK253106	Santos et al. (2020)
*Neo.eucalypti*	PA4	*Eucalyptus* sp.	Brazil	*_*	MK286943	MK253107	Santos et al. (2020)
*Neo.eucalypticola*	CBS 264.37^T^	*Eucalyptusglobulus*	Unknown	KM199376	KM199431	KM199551	Maharachchikumbura et al. (2014b)
*Neo.eucalyptorum*	MEAN 1308 = CBS 147684^T^	*Eucalyptusglobulus*	Portugal	MW794108	MW802841	MW805397	Diogo et al. (2021)
*Neo.eucalyptorum*	MEAN 1323	*Eucalyptusglobulus*	Portugal	MW794099	MW802832	MW805412	Diogo et al. (2021)
*Neo.eucalyptorum*	MEAN 1324	*Eucalyptusglobulus*	Portugal	MW794100	MW802833	MW805413	Diogo et al. (2021)
*Neo.fijiensis*	CGMCC 3.23465 = LC0652 = ICMP6030 Q^T^	*Arachishypogaea*	Fiji	OR247892	OR381003	OR361403	Razaghi et al. (2024)
*Neo.fijiensis*	LC15864	*Arachishypogaea*	Fiji	OR247864	OR381004	OR361404	Razaghi et al. (2024)
*Neo.fimbriata*	CGMCC 3.23479 = LC13340^T^	*Cinnamomumcamphora*	China	OR247869	OR381020	OR361420	Razaghi et al. (2024)
*Neo.fimbriata*	LC0141	Unknown	China	OR247893	OR381002	OR361402	Razaghi et al. (2024)
*Neo.fimbriata*	LC6309	Unknown	China	OR247882	OR381037	OR361437	Razaghi et al. (2024)
*Neo.fimbriata*	LC6285	*Camelliasinensis*	China	KX895013	KX895346	KX895232	Liu et al. (2017)
*Neo.foedans*	CGMCC 3.9123^T^	Mangrove plant leaves	China	JX398987	JX399022	JX399053	Maharachchikumbura et al. (2012)
*Neo.foedans*	CGMCC 3.9178	*Neodypsisdecaryi*	China	JX398989	JX399024	JX399055	Maharachchikumbura et al. (2012)
*Neo.formicarum*	CBS 362.72^T^	Dead Formicidae (ant)	Ghana	KM199358	KM199455	KM199517	Maharachchikumbura et al. (2014b)
*Neo.formicarum*	CBS 115.83	Plant debris	Cuba	KM199344	KM199444	KM199519	Maharachchikumbura et al. (2014b)
*Neo.fragariae*	ZHKUCC 22- 0113^T^	*Fragaria×ananassa*	China	ON553410	ON569075	ON569076	Prematunga et al. (2022)
*Neo.fragariae*	ZHKUCC 22- 0114	*Fragaria×ananassa*	China	ON651145	ON685198	ON685196	Prematunga et al. (2022)
*Neo.fuzhouensis*	CGMCC 3.23509 = LC8457^T^	*Acerbuergerianum*	China	OR247877	OR381047	OR361447	Razaghi et al. (2024)
*Neo.fuzhouensis*	LC15861	*Acerbuergerianum*	China	OR247865	OR381048	OR361448	Razaghi et al. (2024)
*Neo.guajavae*	FMBCC 11.1 = FMB0026	On branches of Guava tree	Pakistan	MF783085	MH460871	MH460868	Haq et al. (2021)
*Neo.guajavae*	FMBCC 11.1 = FMB0027	On branches of Guava tree	Pakistan	MF783084	MH460872	MH460869	Haq et al. (2021)
*Neo.guajavicola*	FMBCC 11.4 = FMB0129^T^	On leaves of Guava tree	Pakistan	MH209245	MH460873	MH460870	Haq et al. (2021)
*Neo.guangxiensis*	CGMCC 3.23505 = LC7542^T^	Poaceae sp.	China	OR247881	OR381040	OR361440	Razaghi et al. (2024)
*Neo.guangxiensis*	LC15866	Poaceae sp.	China	OR247863	OR381041	OR361441	Razaghi et al. (2024)
*Neo.guizhouensis*	CGMCC 3.23501 = LC5337^T^	Air, unnamed karst cave	China	OR247883	OR381036	OR361436	Razaghi et al. (2024)
*Neo.guizhouensis*	LC10106	Cave rock	China	OR247876	OR381005	OR361405	Razaghi et al. (2024)
*Neo.hadrolaeliae*	VIC 47180^T^	*Hadrolaeliajongheana*	Brazil	MK454709	MK465120	MK465122	Freitas et al. (2019)
*Neo.hadrolaeliae*	VIC 47181	*Hadrolaeliajongheana*	Brazil	MK454710	MK465121	MK465123	Freitas et al. (2019)
*Neo.haikouensis*	SAUCC 212271^T^	*Ilexchinensis*	China	OK087294	OK104870	OK104877	Zhang et al. (2021)
*Neo.haikouensis*	SAUCC 212272	*Ilexchinensis*	China	OK087295	OK104871	OK104878	Zhang et al. (2021)
*Neo.hispanica*	MEAN 1310 = CBS 147686^T^	*Eucalyptusglobulus*	Portugal	MW794107	MW802840	MW805399	Diogo et al. (2021)
*Neo.hispanica*	MEAN 1311	*Eucalyptusglobulus*	Portugal	MW794106	MW802839	MW805400	Diogo et al. (2021)
*Neo.hispanica*	CAA1059 = MUM 21.36	*Vacciniumcorymbosum*	Portugal	MW969747	MW934610	MW959099	Santos et al. (2022)
*Neo.hispanica*	CAA1027	*Vacciniumcorymbosum*	Portugal	MW969746	MW934609	MW959098	Santos et al. (2022)
*Neo.honoluluana*	CBS 114495^T^	*Telopea* sp.	USA	KM199364	KM199457	KM199548	Maharachchikumbura et al. (2014b)
*Neo.honoluluana*	CBS 111535	*Telopea* sp.	USA	KM199363	KM199461	KM199546	Maharachchikumbura et al. (2014b)
*Neo.hydeana*	MFLUCC 20-0132^T^	*Artocarpusheterophyllus*	Thailand	MW266069	MW251119	MW251129	Huanluek et al. (2021)
*Neo.hydeana*	MFLUCC 20-0133	*Citrus* sp.	Thailand	MW266071	MW251121	MW251131	Huanluek et al. (2021)
*Neo.hyperici*	KUNCC 22-12597^T^	*Hypericummonogynum*	China	OP498010	OP765908	OP713768	Sun et al. (2023)
*Neo.hyperici*	KUNCC 22-12598	*Hypericummonogynum*	China	OP498009	OP737883	OP737880	Sun et al. (2023)
*Neo.hyperici*	CGMCC 3.23504 = LC7093	*Musabasjoo*	China	OR247907	OR381038	OR361438	Razaghi et al. (2024)
*Neo.hyperici*	LC15859	*Musabasjoo*	China	OR247908	OR381039	OR361439	Razaghi et al. (2024)
*Neo.iberica*	MEAN 1313 = CBS 147688^T^	*Eucalyptusglobulus*	Portugal	MW794111	MW802844	MW805402	Diogo et al. (2021)
*Neo.iberica*	MEAN 1314 = CBS 147689	*Eucalyptusglobulus*	Spain	MW794114	MW802847	MW805403	Diogo et al. (2021)
*Neo.iranensis*	CBS 137768^T^	*Fragariaananassa*	Iran	OR230041	OR381098	OR380984	Razaghi et al. (2024)
*Neo.javaensis*	CBS 257.31	*Cocosnucifera*	Indonesia	KM199357	KM199437	KM199543	Maharachchikumbura et al. (2014b)
*Neo.javaensis*	MFLUCC 12-0594	*Vitisvinifera*	France	KX816905	KX816933	KX816874	Maharachchikumbura et al. (2014b)
*Neo.jiangxiensis*	CGMCC 3.23492 = LC4209^T^	*Rhododendronlatoucheae*	China	OR247890	OR381024	OR361424	Razaghi et al. (2024)
*Neo.jiangxiensis*	LC4210	*Rhododendronlatoucheae*	China	OR247889	OR381025	OR361425	Razaghi et al. (2024)
*Neo.jiangxiensis*	LC4259	*Rhododendronlatoucheae*	China	OR783483	OR792184	OR792183	Razaghi et al. (2024)
*Neo.keteleeriae*	MFLUCC 13-0915^T^	*Keteleeriapubescens*	China	KJ023087	KJ023088	KJ023089	Song et al. (2014)
*Neo.keteleeriae*	GUCC 21501	*Rhapisexcelsa*	China	MW931620	MW980441	MW980442	Yang et al. (2021)
*Neo.liquidambaris*	CGMCC 3.23508 = LC8381^T^	*Liquidambarformosana*	China	OR247878	OR381046	OR361446	Razaghi et al. (2024)
*Neo.liquidambaris*	LC5236	Unknown	China	OR247884	OR381035	OR361435	Razaghi et al. (2024)
***Neo.litseae***	**CGMCC 3.28543^T^**	***Litseaverticillata***	**China**	**PQ681332**	**PQ687596**	**PQ687590**	**This study**
***Neo.litseae***	**CGMCC 3.28544**	***Litseaverticillata***	**China**	**PQ681337**	**PQ687597**	**PQ687591**	**This study**
*Neo.longiappendiculata*	MEAN 1315 = CBS 147690^T^	*Eucalyptusglobulus*	Portugal	MW794112	MW802845	MW805404	Diogo et al. (2021)
*Neo.longiappendiculata*	MEAN 1316 = CBS 147691	*Eucalyptusnitens*	Portugal	MW794103	MW802836	MW805405	Diogo et al. (2021)
***Neo.longqishanensis***	**CGMCC 3.28545^T^**	**Unknown**	**China**	**PQ681338**	**PQ687598**	**PQ687592**	**This study**
***Neo.longqishanensis***	**CGMCC 3.28546**	**Unknown**	**China**	**PQ681339**	**PQ687599**	**PQ687593**	**This study**
*Neo.lusitanica*	MEAN 1317 = CBS 147692^T^	*Eucalyptusglobulus*	Portugal	MW794110	MW802843	MW805406	Diogo et al. (2021)
*Neo.lusitanica*	MEAN 1318 = CBS147693^T^	*Eucalyptusglobulus*	Portugal	MW794093	MW802826	MW805407	Diogo et al. (2021)
*Neo.macadamiae*	BRIP 63737c = CBS 142767^T^	*Macadamiaintegrifolia*	Australia	KX186604	KX186654	KX186627	Akinsanmi et al. (2017)
*Neo.macadamiae*	BRIP 63742a	*Macadamiaintegrifolia*	Australia	KX186599	KX186657	KX186629	Akinsanmi et al. (2017)
*Neo.machili*	CGMCC 3.23477 = LC13302^T^	*Machilusyunnanensis*	China	OR247870	OR381016	OR361416	Razaghi et al. (2024)
*Neo.machili*	LC15848	*Machilusyunnanensis*	China	OR247868	OR381017	OR361417	Razaghi et al. (2024)
*Neo.maddoxii*	BRIP 72266a^T^	*Macadamiaintegrifolia*	Australia	MZ303782	MZ312675	MZ344167	Prasannath et al. (2021)
*Neo.maddoxii*	BRIP 72260a	*Macadamiaintegrifolia*	Australia	MZ303780	MZ312673	MZ344165	Prasannath et al. (2021)
*Neo.maddoxii*	BRIP 72262a	*Macadamiaintegrifolia*	Australia	MZ303781	MZ312674	MZ344166	Prasannath et al. (2021)
*Neo.magna*	MFLUCC 12-0652^T^	*Pteridium* sp.	France	KF582795	KF582793	KF582791	Maharachchikumbura et al. (2014a)
*Neo.megabetaspora*	CGMCC 3.23474 = LC13119^T^	Poaceae sp.	China	OR247875	OR381010	OR361410	Razaghi et al. (2024)
*Neo.megabetaspora*	LC13142	*Brachiaria* sp.	China	OR247873	OR381012	OR361412	Razaghi et al. (2024)
*Neo.megabetaspora*	LC13133	Poaceae sp.	China	OR247874	OR381011	OR361411	Razaghi et al. (2024)
*Neo.mesopotamica*	CBS 336.86^T^	*Pinusbrutia*	Iraq	KM199362	KM199441	KM199555	Maharachchikumbura et al. (2014b)
*Neo.mesopotamica*	CBS 299.74	*Eucalyptus* sp.	Turkey	KM199361	KM199435	KM199541	Maharachchikumbura et al. (2014b)
*Neo.mianyangensis*	CGMCC 3.23555^T^	*Paeoniasuffruticosa*	China	OP546681	OP672161	OP723490	Li et al. (2022)
*Neo.mianyangensis*	UESTCC 22.0006	*Paeoniasuffruticosa*	China	OP082291	OP235979	OP204793	Li et al. (2022)
*Neo.moniliformis*	CGMCC 3.23498 = LC4495^T^	*Phyllostachys* sp.	China	OR247886	OR381031	OR361431	Razaghi et al. (2024)
*Neo.moniliformis*	LC15853	*Phyllostachys* sp.	China	OR247867	OR381032	OR361432	Razaghi et al. (2024)
*Neo.musae*	MFLUCC 15-0776^T^	*Musa* sp.	Thailand	KX789683	KX789686	KX789685	Hyde et al. (2016)
*Neo.nanningensis*	CGMCC 3.23475 = LC13212^T^	*Ixorachinensis*	China	OR247872	OR381014	OR361414	Razaghi et al. (2024)
*Neo.nanningensis*	LC13213	*Ixorachinensis*	China	OR247871	OR381015	OR361415	Razaghi et al. (2024)
*Neo.natalensis*	CBS 138.41^T^	*Acaciamollissima*	South Africa	KM199377	KM199466	KM199552	Maharachchikumbura et al. (2014b)
*Neo.nebuloides*	BRIP 66617^T^	*Sporoboluselongatus*	Australia	MK966338	MK977632	MK977633	Crous et al. (2020)
*Neo.olumideae*	BRIP 72273a^T^	*Macadamiaintegrifolia*	Australia	MZ303790	MZ312683	MZ344175	Prasannath et al. (2021)
*Neo.olumideae*	BRIP 72283a	*Macadamiaintegrifolia*	Australia	MZ303791	MZ312684	MZ344176	Prasannath et al. (2021)
*Neo.paeonia*	CBS 318.74	*Anacardiumoccidentale*	Nigeria	MH554031	MH554707	*_*	Liu et al. (2019)
*Neo.paeonia-suffruticosa*	CGMCC 3.23554^T^	*Paeoniasuffruticosa*	China	OP082292	OP235980	OP204794	Liu et al. (2019)
*Neo.paeonia-suffruticosa*	UESTCC 22.0033	*Paeoniasuffruticosa*	China	OP082293	OP235981	OP204795	Liu et al. (2019)
*Neo.pandanicola*	KUMCC 17-0175^T^	*Pandanus* sp.	China	*_*	MH412720	MH388389	Tibpromma et al. (2018)
*Neo.pernambucana*	URM 7148-01^T^	*Vismiaguianensis*	Brazil	KJ792466	*_*	KU306739	Silvério et al. (2016)
*Neo.pernambucana*	URM 7148-02	*Vismiaguianensis*	Brazil	KJ792467	*_*	KU306740	Silvério et al. (2016)
*Neo.perukae*	FMBCC 11.3 = FMB0127	Fruit of Guava tree	Pakistan	MH209077	MH460876	MH523647	Haq et al. (2021)
*Neo.perukae*	FMBCC 11.3 = FMB0128	Fruit of Guava tree	Pakistan	MH209246	MH460875	MH523646	Haq et al. (2021)
*Neo.perukae*	FMBCC 11.3 = FMB0130	Branches of Guava tree	Pakistan	MH208973	MH477871	MH523648	Haq et al. (2021)
*Neo.petila*	MFLUCC 17-1737^T^	*Rhizophoramucronata*	Thailand	MK764275	MK764341	MK764319	Norphanphoun et al. (2019)
*Neo.petila*	MFLUCC 17-1738	*Rhizophoramucronata*	Thailand	MK764276	MK764342	MK764320	Norphanphoun et al. (2019)
*Neo.phangngaensis*	MFLUCC 18-0119^T^	*Pandanus* sp.	Thailand	MH388354	MH412721	MH388390	Tibpromma et al. (2018)
*Neo.photiniae*	MFLUCC 22-0129^T^	*Photiniaserrulat*	China	OP498008	OP752131	OP753368	Sun et al. (2023)
*Neo.photiniae*	GUCC 21-0820	*Photiniaserrulat*	China	OP806524	OP896200	OP828691	Sun et al. (2023)
*Neo.phyllostachydis*	CGMCC 3.23491 = LC4208^T^	*Phyllostachys* sp.	China	OR247891	OR381023	OR361423	Razaghi et al. (2024)
*Neo.phyllostachydis*	LC4371	*Rhododendronarboreum*	China	OR247887	OR381029	OR361429	Razaghi et al. (2024)
*Neo.phyllostachydis*	LC4225	*Castanopsis* sp.	China	OR247888	OR381027	OR361427	Razaghi et al. (2024)
*Neo.piceana*	CBS 394.48^T^	*Picea* sp.	UK	KM199368	KM199453	KM199527	Maharachchikumbura et al. (2014b)
*Neo.piceana*	CBS 254.32	*Cocosnucifera*	Indonesia	KM199372	KM199452	KM199529	Maharachchikumbura et al. (2014b)
*Neo.poae*	CGMCC 3.23506 = LC7551^T^	Poaceae sp.	China	OR247880	OR381042	OR361442	Razaghi et al. (2024)
*Neo.poae*	LC7562	Poaceae sp.	China	OR247879	OR381043	OR361443	Razaghi et al. (2024)
*Neo.protearum*	CBS 114178^T^	*Leucospermumcuneiforme*	Zimbabwe	JN712498	KM199463	KM199542	Maharachchikumbura et al. (2014b)
*Neo.protearum*	CBS 111506	*Leucospermumcuneiforme*	Zimbabwe	MH553959	MH554618	MH554377	Liu et al. (2019)
*Neo.psidii*	FMBCC 11.2 = FMB0028^T^	Branches of Guava tree	Pakistan	MF783082	MH477870	MH460874	Haq et al. (2021)
*Neo.rhizophorae*	MFLUCC 17-1550^T^	*Rhizophoramucronata*	Thailand	MK764277	MK764343	MK764321	Norphanphoun et al. (2019)
*Neo.rhizophorae*	MFLUCC 17-1551	*Rhizophoramucronata*	Thailand	MK764278	MK764344	MK764322	Norphanphoun et al. (2019)
*Neo.rhododendri*	GUCC 21504^T^	*Rhododendronsimsii*	China	MW979577	MW980443	MW980444	Yang et al. (2021)
*Neo.rhododendri*	GUCC 21505	*Rhododendronsimsii*	China	MW979576	MW980445	MW980446	Yang et al. (2021)
*Neo.rhododendricola*	KUN-HKAS 123204^T^	*Rhododendron* sp.	China	OK283069	OK274147	OK274148	Chaiwan et al. (2022)
*Neo.rosae*	CBS 101057^T^	*Rosa* sp.	New Zealand	KM199359	KM199429	KM199523	Maharachchikumbura et al. (2014b)
*Neo.rosae*	CBS 124745	*Paeoniasuffruticosa*	USA	KM199360	KM199430	KM199524	Maharachchikumbura et al. (2014b)
*Neo.rosicola*	CFCC 51992^T^	*Rosachinensis*	China	KY885239	KY885245	KY885243	Jiang et al. (2018)
*Neo.rosicola*	CFCC 51993	*Rosachinensis*	China	KY885240	KY885246	KY885244	Jiang et al. (2018)
*Neo.samarangensis*	MFLUCC 12-0233^T^	*Syzygiumsamarangense*	Thailand	JQ968609	JQ968610	JQ968611	Maharachchikumbura et al. (2014b)
*Neo.samarangensis*	CBS 115451	Unidentified tree	China	KM199365	KM199447	KM199556	Maharachchikumbura et al. (2014b)
*Neo.saprophytica*	MFLUCC 12-0282^T^	*Magnolia* sp.	China	JX398982	JX399017	JX399048	Maharachchikumbura et al. (2012)
*Neo.saprophytica*	CBS 115452	*Litsearotundifolia*	China	KM199345	KM199433	KM199538	Maharachchikumbura et al. (2014b)
*Neo.scalabiensis*	CAA1029 = MUM 21.34^T^	*Vacciniumcorymbosum*	Portugal	MW969748	MW934611	MW959100	Santos et al. (2022)
*Neo.sichuanensis*	CFCC 54338^T^	*Castaneamollissima*	China	MW166231	MW218524	MW199750	Jiang et al. (2021)
*Neo.sichuanensis*	SM15-1C	*Castaneamollissima*	China	MW166232	MW218525	MW199751	Jiang et al. (2021)
*Neo.siciliana*	AC46 = CBS 149117^T^	*Perseaamericana*	Italy	ON117813	ON209162	ON107273	Fiorenza et al. (2022)
*Neo.siciliana*	AC48 = CBS 149118	*Perseaamericana*	Italy	ON117812	ON209163	ON107274	Fiorenza et al. (2022)
*Neo.smilacis*	CGMCC 3.23500 = LC4596^T^	*Smilaxlanceifolia*	China	OR247885	OR381033	OR361433	Razaghi et al. (2024)
*Neo.smilacis*	LC15854	*Smilaxlanceifolia*	China	OR247866	OR381034	OR361434	Razaghi et al. (2024)
*Neo.sonneratae*	MFLUCC 17-1745^T^	*Sonneronataalba*	Thailand	MK764280	MK764346	MK764324	Norphanphounet et al. (2019)
*Neo.sonneratae*	MFLUCC 17-1744	*Sonneronataalba*	Thailand	MK764279	MK764345	MK764323	Norphanphounet et al. (2019)
*Neo.steyaertii*	IMI 192475^T^	*Eucalyptusviminalis*	Australia	KF582796	KF582794	KF582792	Maharachchikumbura et al. (2014a,b)
*Neo.subepidermalis*	CFCC 55160^T^	*Rosarugosa*	China	OK560699	OM117690	OM622425	Peng et al. (2022)
*Neo.subepidermalis*	CFCC 55161	*Rosachinensis*	China	OK560701	OM117692	OM622427	Peng et al. (2022)
*Neo.suphanburiensis*	MFLUCC 22-0126^T^	Unidentified plant	Thailand	OP497994	OP752135	OP753372	Sun et al. (2023)
*Neo.surinamensis*	CBS 450.74^T^	Soil under *Elaeisguineensis*	Suriname	KM199351	KM199465	KM199518	Maharachchikumbura et al. (2014b)
*Neo.surinamensis*	CBS 111494	*Proteaeximia*	Zimbabwe	JX556232	KM199462	KM199530	Maharachchikumbura et al. (2014b)
*Neo.terricola*	CGMCC 3.23553^T^	*Paeoniasuffruticosa*	China	OP082294	OP235982	OP204796	Li et al. (2022)
*Neo.terricola*	UESTCC 22.0034	*Paeoniasuffruticosa*	China	OP082295	OP235983	OP204797	Li et al. (2022)
*Neo.thailandica*	MFLUCC 17-1730^T^	*Rhizophoramucronata*	Thailand	MK764281	MK764347	MK764325	Norphanphoun et al. (2019)
*Neo.thailandica*	MFLUCC 17-1731	*Rhizophoramucronata*	Thailand	MK764282	MK764348	MK764326	Norphanphoun et al. (2019)
*Neo.umbrinospora*	MFLUCC 12-0285^T^	Unidentified plant	China	JX398984	JX399019	JX399050	Maharachchikumbura et al. (2012)
*Neo.vacciniicola*	CAA1055 = MUM 21.35^T^	*Vacciniumcorymbosum*	Portugal	MW969751	MW934614	MW959103	Santos et al. (2022)
*Neo.vacciniicola*	CAA1054	*Vacciniumcorymbosum*	Portugal	MW969750	MW934613	MW959102	Santos et al. (2022)
*Neo.vheenae*	BRIP 72293a^T^	*Macadamiaintegrifolia*	Australia	MZ303792	MZ312685	MZ344177	Prasannath et al. (2021)
*Neo.vitis*	MFLUCC 15-1265^T^	*Vitisvinifera*	China	KU140694	KU140685	KU140676	Jayawardena et al. (2016)
*Neo.vitis*	MFLUCC 15-1270	*Vitisvinifera*	China	KU140699	KU140690	KU140681	Jayawardena et al. (2016)
*Neo.vitis*	CBS 266.80	*Vitisvinifera*	India	KM199352	*_*	KM199532	Jayawardena et al. (2016)
*Neo.zakeelii*	BRIP 72282a^T^	*Macadamiaintegrifolia*	Australia	MZ303789	MZ312682	MZ344174	Prasannath et al. (2021)
*Neo.zakeelii*	BRIP 72271a	*Macadamiaintegrifolia*	Australia	MZ303788	MZ312681	MZ344173	Prasannath et al. (2021)
*Neo.zimbabwana*	CBS 111495^T^	*Leucospermumcuneiforme*	Zimbabwe	JX556231	KM199456	KM199545	Maharachchikumbura et al. (2014b)
*Neo.zingiberis*	HGUP10001^T^	*Zingiberofficinale*	China	MW930715	MZ683390	MZ683389	He et al. (2022)
*Neo.zingiberis*	HGUP10005	*Zingiberofficinale*	China	ON597078	ON595538	ON595536	He et al. (2022)
*Neopestalotiopsis* sp.	MEAN 1325	*Eucalyptusglobulus*	Portugal	MW794102	MW802835	MW805414	Diogo et al. (2021)
*Neopestalotiopsis* sp.	MEAN 1327	*Eucalyptusglobulus*	Portugal	MW794105	MW802838	MW805416	Diogo et al. (2021)
*Neopestalotiopsis* sp.	MEAN 1328	*Eucalyptusglobulus*	Spain	MW794115	MW802848	MW805417	Diogo et al. (2021)
*Neopestalotiopsis* sp.	CFCC 54337	*Castaneamollissima*	China	MW166233	MW218526	MW199752	Jiang et al. (2021)
*Neopestalotiopsis* sp.	ZX12-1	*Castaneamollissima*	China	MW166234	MW218527	MW199753	Jiang et al. (2021)
*Neopestalotiopsis* sp.	CFCC 54340	*Castaneamollissima*	China	MW166235	MW218528	MW199754	Jiang et al. (2021)
*Neopestalotiopsis* sp.	ZX22B	*Castaneamollissima*	China	MW166236	MW218529	MW199755	Jiang et al. (2021)
*Neopestalotiopsis* sp.	CSUFTCC61	*Camelliaoleifera*	China	OK493590	OK562365	OK507960	Li et al. (2021)
*Neopestalotiopsis* sp.	CSUFTCC62	*Camelliaoleifera*	China	OK493591	OK562366	OK507961	Li et al. (2021)
*Neopestalotiopsis* sp.	CSUFTCC63	*Camelliaoleifera*	China	OK493592	OK562367	OK507962	Li et al. (2021)
*Neopestalotiopsis* sp.	CBS 233.79	*Crotalariajuncea*	India	KM199373	KM199464	KM199528	Maharachchikumbura et al. (2014b)
*Neopestalotiopsis* sp.	CBS 664.94	*Cocosnucifera*	Netherlands	KM199354	KM199449	KM199525	Maharachchikumbura et al. (2014b)
*Neopestalotiopsis* sp.	CBS 177.25	*Dalbergia* sp.	Unknown	KM199370	KM199445	KM199533	Maharachchikumbura et al. (2014b)
*Neopestalotiopsis* sp.	CBS 266.37 = BBA 5087 = IMI 083708	*Erica* sp.	Germany	KM199349	KM199459	KM199547	Maharachchikumbura et al. (2014b)
*Neopestalotiopsis* sp.	CBS 361.61	*Cissus* sp.	Netherlands	KM199355	KM199460	KM199549	Maharachchikumbura et al. (2014b)
*Neopestalotiopsis* sp.	CBS 323.76	*Ericagracilis*	France	KM199350	KM199458	KM199550	Maharachchikumbura et al. (2014b)
*Neopestalotiopsis* sp.	CBS 119.75	*Achrassapota*	India	KM199356	KM199439	KM199531	Senanayake et al. (2020)
*Neopestalotiopsis* sp.	CGMCC 3.23473 = LC1310 = MFLUCC 2010-0901	Para rubber leaf litter	Thailand	OR247910	OR381009	OR361409	Razaghi et al. (2024)
*Neopestalotiopsis* sp.	LC1321 = MFLUCC 2010-0902	Para rubber leaf litter	Thailand	OR247909	OR381013	OR361413	Razaghi et al. (2024)
*Pestalotiopsisdiversiseta*	MFLUCC 12-0287^T^	*Rhododendron* sp.	China	NR_120187	JX399040	JX399073	Maharachchikumbura et al. (2012)
*Pestalotiopsiscolombiensis*	CBS 118553^T^	*Eucalyptus* sp.	Colombia	KM199307	KM199421	KM199488	Maharachchikumbura et al. (2014b)

Notes: Ex-type strains are labelled with ^T^. The strains in this study are indicated in bold.

### ﻿Phylogenetic analyses

NCBI-BLAST searches using sequence data generated from fungal samples were used to identify and download orthologous sequences from GenBank for multi-locus phylogenetic analyses (Tables 2, 3). Gene sequences were initially aligned with MAFFT v.7 and edited manually with MEGA 7.0.20 software and trimAL v.1.2 (http://trimal.cgenomics.org, accessed on 20 October 2024) (Kumar et al. 2016; Katoh et al. 2019; Lei et al. 2023). Multi-locus phylogenetic analyses of the concatenated aligned dataset were obtained by Maximum Likelihood (ML) and Bayesian Inference (BI) methods and inferred using IQtree 1.6.8 (Nguyen et al. 2015) and MrBayes 3.2.6 (Ronquist et al. 2012) with Phylosuite software v.1.2.3 (Xiang et al. 2023). For the ML analysis, Maximum-Likelihood phylogenies were inferred using IQ-TREE under best partitioned models and tree stability was evaluated with 5000 ultrafast bootstraps (Hoang et al. 2017). For the BI analysis, Bayesian Inference phylogenetic trees were constructed using MrBayes 3.2.6. PartitionFinder2 was used to select the best-fit partition model (Lanfear et al. 2017). A total of two Markov chains were simultaneously run for 2 million generations beginning with a random tree and sampling was conducted every 100 generations. The first 25% of sampled trees were discarded as burn-in and the remaining trees were used to calculate posterior probabilities (PP). The phylogenetic trees were visualised using FigTree 1.4.4 (http://tree.bio.ed.ac.uk/software/figtree, accessed on 20 October 2024) and embellished with Adobe Illustrator CS 6.0 (Adobe Systems Inc., San Jose, CA, USA).

**Table 3.** Speciesnames, strain number, substrate or host, locations, and corresponding GenBank accession numbers of DNA sequences used in the molecular phylogenetic analyses of *Pseudopestalotiopsis*.

Species	Specimen voucher /Strain	Host/Substrate	Locations	GenBank Accession Number			References
				ITS	*tub2*	*tef1*	
*Pseudopestalotiopsisampullacea*	LC6618^T^	*Camelliasinensis*	China	KX895025	KX895358	KX895244	Liu et al. (2017)
*Ps.annellata*	NTUCC 17-030^T^	*Camelliasinensis*	China, Taiwan	MT322087	MT321889	MT321988	Tsai et al. (2018)
*Ps.avicenniae*	MFLUCC 17-0434^T^	*Avicenniamarina*	Thailand	MK764287	MK764353	MK764331	Norphanphoun et al. (2019)
*Ps.avicenniae*	LF48-0709	*Alpiniaoxyphylla*	China	PP621744	PP767825	PP767861	Cui et al. (2024)
*Ps.camelliae*	CGMCC 3.9192	*Camelliasinensis*	China	*_*	KU562851	KU562850	Maharachchikumbura et al. (2016)
*Ps.camelliae-sinensis*	NTUCC 18-031	*Camelliasinensis*	China, Taiwan	MT322047	MT321849	MT321948	Tsai et al. (2018)
*Ps.camelliae-sinensis*	LC3490^T^	*Camelliasinensis*	China	KX894985	KX895316	KX895202	Liu et al. (2017)
*Ps.chinensis*	NTUCC 18-066	*Camelliasinensis*	China, Taiwan	MT322083	MT321885	MT321984	Tsai et al. (2018)
*Ps.chinensis*	LC3011^T^	*Camelliasinensis*	China	KX894937	KX895269	KX895154	Liu et al. (2017)
*Ps.chinensis*	NTUCC 18-038	*Camelliasinensis*	China, Taiwan	MT322055	MT321857	MT321956	Tsai et al. (2018)
*Ps.cocos*	CBS 272.29^T^	*Cocosnucifera*	Indonesia	MH855069	KM199467	KM199553	Maharachchikumbura et al. (2012)
*Ps.celtidis*	GUCC 21599^T^	*Celtissinensis*	China	OL423535	OL439010	OL439012	Yang et al. (2022)
*Ps.curvatispora*	MFLUCC 17-1723	*Rhizophoramucronata*	Thailand	MK764290	MK764356	MK764334	Norphanphoun et al. (2019)
*Ps.curvatispora*	MFLUCC 17-1722^T^	*Rhizophoramucronata*	Thailand	MK764289	MK764355	MK764333	Norphanphoun et al. (2019)
*Ps.dawaina*	INPA 2912	*Caryotamitis*	Brazil	MN096659	MN151310	MN151308	Catarino et al. (2020)
*Ps.dawaina*	MM14-F0015^T^	Unknown	Dawei, Myanmarr	LC324750	LC324751	LC324752	Nozawa et al. (2018)
*Ps.elaeidis*	CBS 413.62^T^	*Elaeisguineensis*	Nigeria	MH554044	MH554720	MH554479	Liu et al. (2019)
*Ps.elaeidis*	CBS 144023	*Acaciacrassipes*	Indonesia	MH554106	MH554779	MH554540	Liu et al. (2019)
*Ps.gilvanii*	INPA 2913^T^	*Paulliniacupana*	Brazil	MN385951	MN385954	MN385957	Gualberto et al. (2021)
*Ps.hydeae*	NTUCC 17-003.1	*Diospyros* sp.	China, Taiwan	MG816313	MG816323	MG816333	Tsai et al. (2018)
*Ps.ignota*	NN 42909^T^	*Camelliasinensis*	China	KU500020	*_*	KU500016	Maharachchikumbura et al. (2016)
*Ps.indica*	CBS 459.78^T^	*Hibiscusrosa-sinensis*	India	KM199381	KM199470	KM199560	Maharachchikumbura et al. (2014)
*Ps.indocalami*	GUCC 21600^T^	*Indocalamustessellatus*	China	OL423536	OL439011	OL439013	Yang et al. (2022)
*Ps.ixorae*	NTUCC 17-001.1^T^	*Lxora* sp.	Unknown	MG816316	MG816326	MG816336	Tsai et al. (2018)
*Ps.kawthaungina*	MM14F0083^T^	Unknown	Kawthaung, Myanmar	LC324753	LC324754	LC324755	Nozawa et al. (2018)
*Ps.kubahensis*	UMAS-KUB-P20^T^	*Macaranga* sp.	Sarawak, Malaysia	MG818971	*_*	*_*	Lateef et al. (2015)
*Ps.myanmarina*	NBRC 112264^T^	*Averrhoacarambola*	Dawei, Myanmarr	LC114025	LC114045	LC114065	Nozawa et al. (2017)
*Ps.myanmarina*	JR34-0709	*Alpiniaoxyphylla*	China	PP621737	PP767824	PP767860	Cui et al. 2024
*Ps.rhizophorae*	MFLUCC 17-1560^T^	*Rhizophoraapiculata*	Thailand	MK764291	MK764357	MK764335	Norphanphoun et al. (2019)
*Ps.simitheae*	KUMCC 17-0255	*Magnoliacandolli*	China	MW244023	MW602387	MW273930	Silva et al. (2021)
*Ps.simitheae*	MFLUCC 12-0121^T^	*Pandanusodoratissimus*	Thailand	KJ503812	KJ503815	KJ503818	Song et al. (2014)
*Ps.solicola*	CBS 386.97^T^	Soil in tropical forest	Papua New Guinea	MH554039	MH554715	MH554474	Liu et al. (2019)
*Ps.taiwanensis*	NTUCC 17-002.1^T^	*Ixora* sp.	China, Taiwan	MG816319	MG816329	MG816339	Tsai et al. (2018)
*Ps.thailandica*	MFLUCC 17-1724^T^	*Rhizophoramucronata*	Thailand	MK764292	MK764358	MK764336	Norphanphoun et al. (2019)
*Ps.thailandica*	MFLUCC 17-1725	*Rhizophoramucronata*	Thailand	MK764293	MK764359	MK764337	Norphanphoun et al. (2019)
*Ps.theae*	MFLUCC 12-0055^T^	*Camelliasinensis*	Thailand	JQ683727	JQ683711	JQ683743	Maharachchikumbura et al. (2012)
*Ps.theae*	SC011	*Camelliasinensis*	Thailand	JQ683726	JQ683710	JQ683742	Maharachchikumbura et al. (2014b)
*Ps.vietnamensis*	NBRC 112252	*Fragaria* sp.	Hue, Vietnam	LC114034	LC114054	LC114074	Nozawa et al. (2017)
***Ps.zhangzhouensis***	**CGMCC 3.28547^T^**	***Ixorachinensis***	**China**	**PQ681341**	**PQ687600**	**PQ687594**	**This study**
***Ps.zhangzhouensis***	**CGMCC 3.28548**	***Ixorachinensis***	**China**	**PQ681342**	**PQ687601**	**PQ687595**	**This study**
*Pestalotiopsistrachycarpicola*	OP068^T^	*Trachycarpusfortunei*	China	JQ845947	JQ845945	JQ845946	Zhang et al. (2012)

Notes: Ex-type strains are labelled with ^T^. The strains in this study are indicated in bold.

## ﻿Results

### ﻿Phylogenetic analyses of *Neopestalotiopsis* and *Pseudopestalotiopsis*

**Figure 1.**
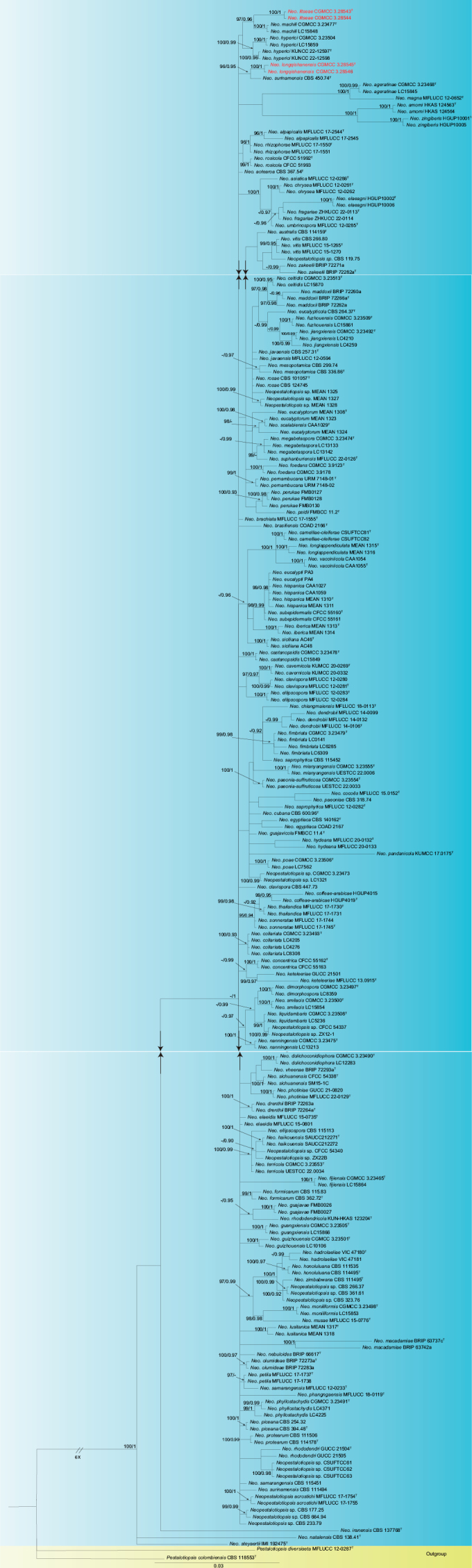
Phylogenetic relationship of *Neopestalotiopsis*, based on concatenated sequences of ITS, *tub2* and *tef1* sequence data. Branch support values are indicated above the nodes as ML bootstrap supports (≥ 95%) and BI posterior probabilities (≥ 0.90). The tree is rooted to *Pestalotiopsisdiversiseta* (MFLUCC 12-0287) and *Pestalotiopsiscolombiensis* (CBS 118553). Novel species are in red and “T” indicates the type specimen. Some branches are shortened according to the indicated multipliers to fit the page size and these are indicated by the symbol (//).

**Figure 2.**
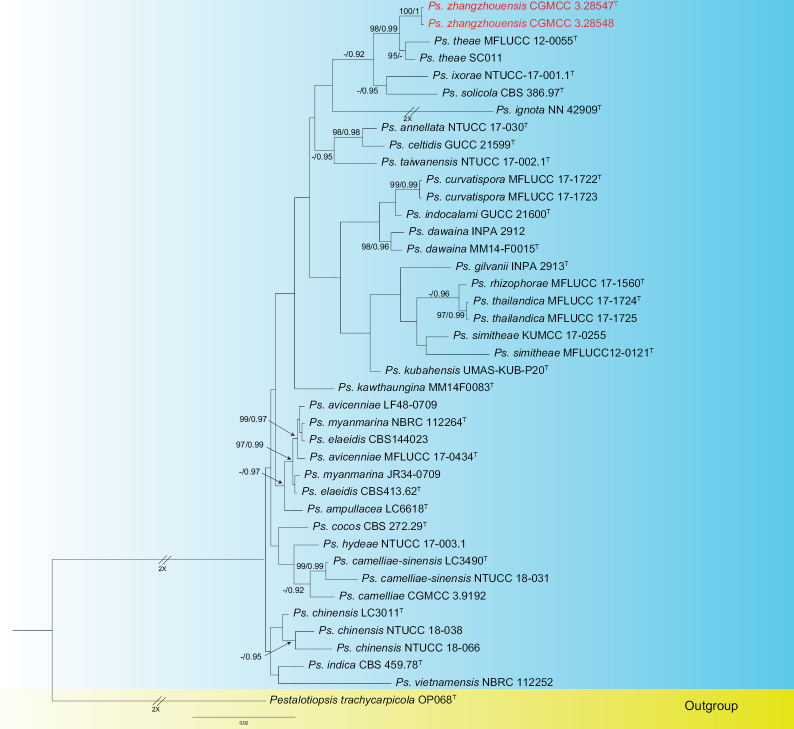
Phylogenetic relationship of *Pseudopestalotiopsis*, based on concatenated sequences of ITS, *tub2* and *tef1* sequence data. Branch support values are indicated above the nodes as ML bootstrap supports (≥ 95%) and BI posterior probabilities (≥ 0.90). The tree is rooted to *Pestalotiopsistrachycarpicola* (OP068). Novel species are in red and “T” indicates the type specimen. Some branches are shortened according to the indicated multipliers to fit the page size and these are indicated by the symbol (//).

For *Neopestalotiopsis* and *Pseudopestalotiopsis*, the ITS, *tub2* and *tef1* sequence datasets were used to construct phylogenetic trees. For *Neopestalotiopsis*, *Pestalotiopsisdiversiseta* (MFLUCC 12-0287) and *Pestalotiopsiscolombiensis* (CBS 118553) are used as outgroups (Fig. 1). The aligned three-loci dataset had an alignment length of 1892 total characters (ITS: 1–604, *tub2*: 605–1346, *tef1*: 1347–1892). The best model for the dataset was estimated by PartitionFinder2 and Bayesian analysis selected the ITS model as GTR + I + G (Lsetnst = 6, rates = invgamma), the *tub2* model as GTR + I + G (Lsetnst = 6, rates = invgamma), the *tef1* model as GTR + I + G (Lsetnst = 6, rates = invgamma). The Bayesian analysis resulted in a mean standard deviation of split frequencies = 0.008685. For *Pseudopestalotiopsis*, *Pestalotiopsistrachycarpicola* (OP068) is used as the outgroup (Fig. 2). The dataset had an alignment length of 1791 total characters (ITS: 1–569, *tub2*: 570–1300, *tef1*: 1301–1791). The best model for the dataset was estimated by PartitionFinder2 and Bayesian analysis selected the ITS model as HKY + I + G (Lsetnst = 2, rates = invgamma), the *tub2* model as GTR + G (Lsetnst = 6, rates = gamma) and *tef1* modelled as HKY + G (Lsetnst = 2, rates = gamma). The Bayesian analysis resulted in a mean standard deviation of split frequencies = 0.003261. The topology of the ML tree was similar to the Bayesian dervived tree; thus, only the Bayesian tree is shown (Figs 1, 2).

## ﻿Taxonomy

### ﻿ Neopestalotiopsis litseae

Taxon classificationFungiAmphisphaerialesSporocadaceae

Z.A. Heng & J.Z. Qiu sp. nov.

1B7F61EB-8648-5766-8820-682E803138ED

856857

Fig. 3

#### Type.

China • Fujian Province: Zhangzhou City, 24°30'36"N, 117°39'0"E, on diseased leaves of *Litseaverticillata*, September 2023, Z.A. Heng, holotype HMAS 353367; ex-holotype culture CGMCC 3.28543. China • Fujian Province: Zhangzhou City, 24°30'36"N, 117°39'0"E, on diseased leaves of *Litseaverticillata*, September 2023, Z.A. Heng, paratype HMAS 353368; ex-paratype culture CGMCC 3.28544.

**Figure 3.**
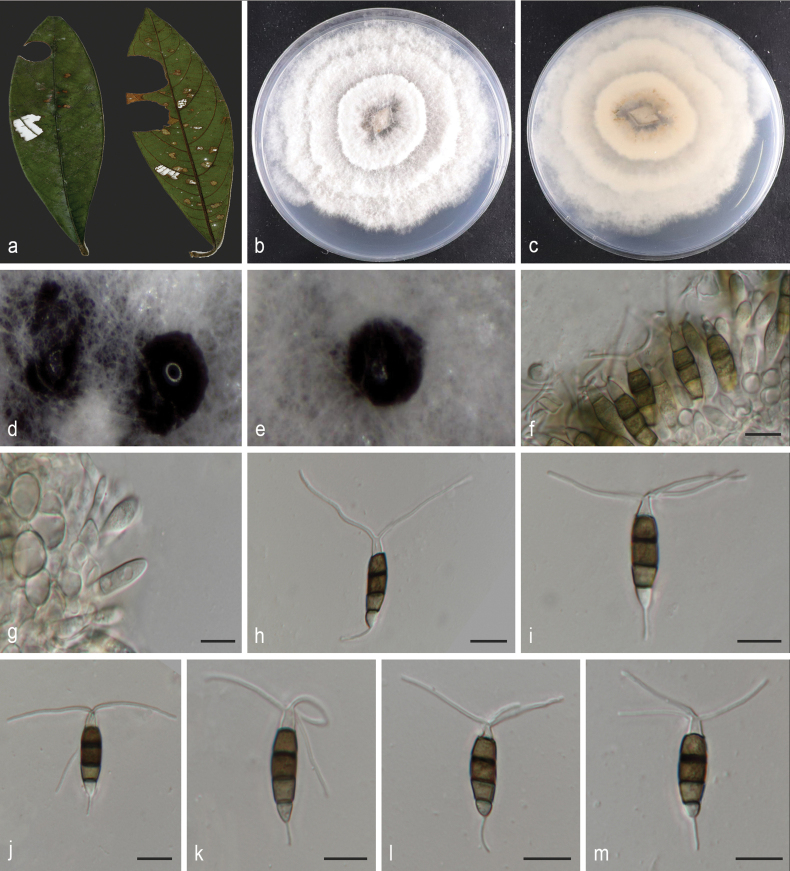
*Neopestalotiopsislitseae* (holotype HMAS 353367) **a** leaves of host shrub **b, c** colony on PDA after 7 days (surface and reverse) **d, e** conidiomata on PDA **f–g** conidiogenous cells and conidia **h–m** conidia. Scale bars: 10 µm (**f–m**).

#### Etymology.

Referring to the host genus from which it was isolated, *Litseaverticillata*.

#### Diagnosis.

Asexual morph on PDA: Conidiomata spherical or hemispherical submerged on PDA, black conidiophores hyaline, rugose and thin-walled, often reduced to conidiogenous cells. Conidia columnar, straight or slightly curved, 19.2–27.7 × 4.6–6.8 μm (mean = 23.4 × 5.8 μm); 4–septate, basal cells obconic to narrowly obconic, 3.2–5.7 μm (mean = 4.0 μm) long, hyaline, thin- and smooth-walled; the three intermediate cells columnar, versicoloured, septa darker than the rest of cells, 12.2–17.2 μm (mean = 15.2 μm) long; the second cell from the base light brown, 3.2–5.9 μm (mean = 4.7 μm) long; the third and fourth cells are dark brown; the third cell 4.3–6.4 μm (mean = 5.1 μm) long; the fourth cell 4.1–6.5 μm (mean = 4.9 μm) long; apical cell hyaline, conical or sub cylindrical, 2–5 μm (mean = 4 μm); with 2–3 tubular apical appendages (mostly 3) arising at different parts of the apical cell, unbranched, filiform, flexuous, 9.3–42.6 μm (mean = 22 μm) long; basal appendages single, tubular, unbranched, 3.2–8.8 μm (mean = 5.9 μm) long. Sexual morph: Unknown.

#### Cultivation characteristics.

Colonies on PDA were nearly circular, grew rapidly, reaching 71–74.5 mm diam. after 7 d at 25 °C; colony initially white, becoming grey-white or pale yellow after 14 days, conidiomata scarce, scattered, black, reverse side of the colony, faint yellow.

#### Notes.

Two isolates corresponding to *Neopestalotiopsislitseae* (CGMCC 3.28543 and CGMCC 3.28544) formed a distinct branch to *Neo.machili* (CGMCC 3.23477 and LC15848) with 97% ML/0.96 BYPP statistical support (Fig. 1). *Neopestalotiopsislitseae* (CGMCC 3.28543) is closely related to *Neo.machili* (CGMCC 3.23477) and comparisons of the nucleotide sequences examined showed 19 bp differences in three loci (3 bp for ITS and 16 bp for *tef1*, including four gaps). *Neo.litseae* is morphologically distinct from *Neo.machili* with narrower conidia 4.6–6.8 μm vs. 7–8.5 μm (Razaghi et al. 2024).

### ﻿ Neopestalotiopsis longqishanensis

Taxon classificationFungiAmphisphaerialesSporocadaceae

Z.A. Heng & J.Z. Qiu sp. nov.

4FC58C77-F4D7-586F-9BE0-8F16B48C1E93

856859

Fig. 4

#### Type.

China • Fujian Province: Longqi Mountain National Nature Reserve, 26°39'28'′ N, 117°51'16'′ E, on diseased leaves of an unknown shrub, September 2023, Z.A. Heng, holotype HMAS 353369; ex-holotype culture CGMCC 3.28545. China • Fujian Province: Longqi Mountain National Nature Reserve, 26°30'27"N, 117°17'47"E, on diseased leaves of an unknown shrub, September 2023, Z.A. Heng, paratype HMAS 353370; ex-paratype culture CGMCC 3.28546.

**Figure 4.**
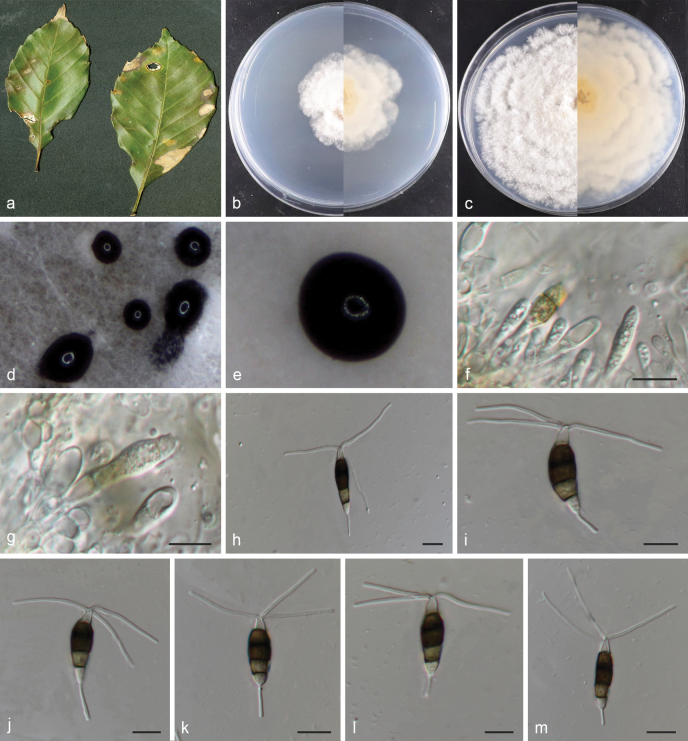
*Neopestalotiopsislongqishanensis* (holotype HMAS 353369) **a** leaves of host shrub **b, c** surface and reverse sides of colony after 7 and 14 d on PDA **d, e** conidiomata on PDA **f–g** conidiogenous cells and conidia **h–m** conidia. Scale bars: 10 µm (**f–m**).

#### Etymology.

Referring to the locality from which it was collected, Longqi Mountain National Nature Reserve.

#### Diagnosis.

Asexual morph on PDA: Conidiomata globose, solitary or aggregated, semi-submerged on PDA, black, conidiophores indistinct, often reduced to conidiogenous cells. Conidiogenous cells hyaline, smooth-walled, cylindrical or jug-shaped. Conidia fusoid, ellipsoid to subcylindrical, straight to slightly curved, 20–24.7 × 4.7–6.7 μm (mean = 22.5 × 5.9 μm); 4–septate; basal cells obconic, 2.5–5.6 μm (mean = 4.1 μm) long, hyaline, thin- and smooth-walled; the three intermediate cells columnar, versicoloured, septa darker than the rest of cells, 12.9–17.6 μm (mean = 15.2 μm) long; the second cell from the base pale brown, 3.7–6.8 μm (mean = 4.8 μm) long; third cell dark brown 3.7–6.5 μm (mean = 5.1 μm) long; fourth cell brown 4.3–6 μm (mean = 4.9 μm) long; apical cell hyaline, conical to subcylindrical, 2.3–5.4 μm (mean = 3.6 μm) long; with 2–3 tubular apical appendages (mostly 3) arising at different parts of the apical cell, unbranched, filiform, flexuous, 18.5–40.7 μm (mean = 27.3 μm) long; basal appendages single, tubular, unbranched, 3.7–10.8 μm (mean = 7.4 μm) long. Sexual morph: Unknown.

#### Cultivation characteristics.

Colonies on PDA attaining 37–41 mm diam. after 7 d at 25 °C, with black conidiomata clusters on the surface. Edges of the colony waved, colony initially white, becoming grey-white after 14 d, reverse side of the colony, pale honey-coloured.

#### Notes.

*Neo.longqishanensis* (CGMCC 3.28545 and CGMCC 3.28546) formed a distinct branch to *Neo.surinamensis* (CBS 450.74 and CBS 111494) with 96% ML/0.95 BYPP statistical support (Fig. 1). The ex-holotype strain *Neo.longqishanensis* (CGMCC 3.28545) is closely related to *Neo.surinamensis* (CBS 450.74) and comparisons of their nucleotides showed 9 bp nucleotide differences in three loci (3 bp for ITS, 5 bp for *tub2* and 1 bp for *tef1*). *Neo.longqishanensis* is morphologically distinct from *Neo.surinamensis* with narrower conidia 4.7–6.7 μm vs. (7–)7.5–9(–9.5) μm and shorter basal cells 2.5–5.6 μm vs. 5–7.5 μm (Maharachchikumbura et al. 2014b).

### ﻿ Pseudopestalotiopsis zhangzhouensis

Taxon classificationFungiAmphisphaerialesSporocadaceae

Z.A. Heng & J.Z. Qiu sp. nov.

0DAB61B3-20FB-5D6E-9EB9-18B5EF49D72B

856860

Fig. 5

#### Type.

China • Fujian Province: Zhangzhou City, 24°30'36"N, 117°39'0"E, on diseased leaves of *Ixorachinensis*, September 2023, Z.A. Heng, holotype HMAS 353371; ex-holotype culture CGMCC 3.28547. China • Fujian Province: Zhangzhou City, 24°30'36"N, 117°39'0"E, on diseased leaves of *Ixorachinensis*, September 2023, Z.A. Heng, paratype HMAS 353372; ex-paratype culture CGMCC 3.28548.

**Figure 5.**
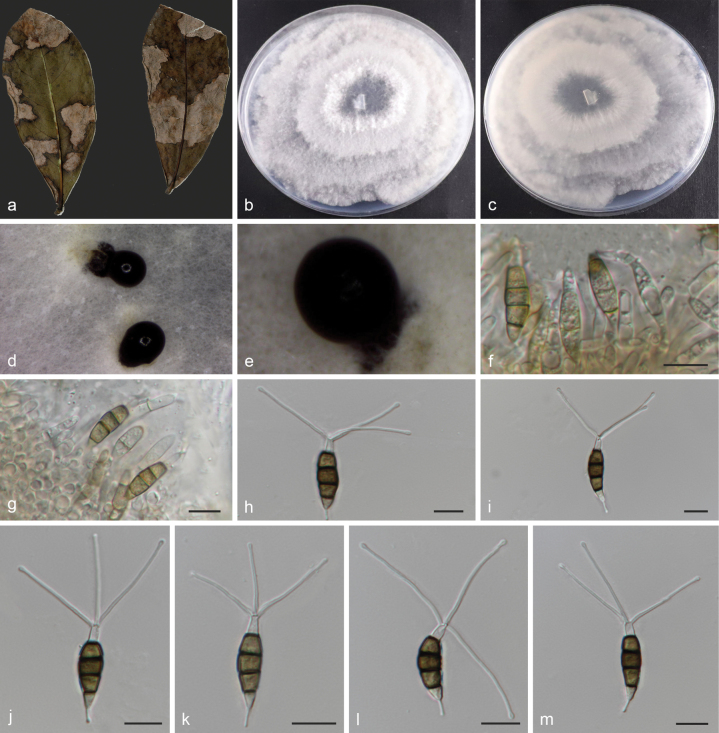
*Pseudopestalotiopsiszhangzhouensis* (holotype HMAS 353371) **a** leaf of host shrub **b, c** colony on PDA after 7 days (above and reverse) **d, e** conidiomata on PDA **f-g** conidiogenous cells and conidia **h–m** conidia. Scale bars: 10 µm (**f–m**).

#### Etymology.

Referring to the locality from which it was collected, China, Fujian Province, Zhangzhou City.

#### Diagnosis.

Asexual morph on PDA: conidiomata acervular, globose, dark brown or black, solitary or aggregated, semi-submerged on PDA, releasing conidia in a black, slimy, globose mass. Conidiophores indistinct and reduced to conidiogenous cells. Conidiogenous cells discrete, smooth-walled, cylindrical or finely lobed. Conidia fusiform, straight or slightly curved, 20.1–27.3 × 4.2–6.9 μm (mean = 23.3 × 5.5 μm); 4–septate, slightly constricted at the septa; basal cells obconical, 2.9–5.2 μm (mean = 4.2 μm) long, hyaline, smooth, thin-walled; the three intermediate cells columnar or cylindrical, homochromatic, pale brown to brown, 13.8–17.9 μm (mean = 15.5 μm) long, septa and periclinal walls darker than rest of the cell; second cell from the base pale brown, 4.1–6.3 μm (mean = 4.9 μm) long; third cell brown, 3.9–5.8 μm (mean = 4.8 μm) long; fourth cell pale brown to brown, 3.9–5.3 μm (mean = 4.5 μm) long; apical cell hyaline, subcylindrical, 2.2–5.1 μm (mean = 3.4 μm) long; with 2–3 tubular apical appendages (mostly 3) arising at different parts of the apical cell, unbranched, 25.1–36.7 μm (mean = 28.5 μm) long; basal appendages single, tubular, unbranched, 2–5.5 μm (mean = 3.9 μm) long. Sexual morph not observed.

#### Cultivation characteristics.

Colonies on PDA grew fast, covering the Petri plate after 7 d of incubation at 25 °C. Colony edges wavy, white or yellowish, with solitary or aggregated clusters of conidiomata on the surface, reverse side of the colony, white.

#### Notes.

Two strains *Pseudopestalotiopsiszhangzhouensis* (CGMCC 3.28547 and CGMCC 3.28548) were isolated from diseased leaf spots on *Ixorachinensis*. *Pseudopestalotiopsiszhangzhouensis* (CGMCC 3.28547 and CGMCC 3.28548) formed a distinct branching relationship to *Ps.theae* (MFLUCC-0055 and SC011) with 98% ML/0.99 BYPP statistical support (Fig. 2). The isolate is closely related to *Ps.theae* (MFLUCC-0055) and comparisons of their nucleotides showed 14 bp nucleotide differences in three loci (6 bp for *tub2* and 8 bp for *tef1*, including two gaps). *Ps.zhangzhouensis* was morphologically distinct from *Ps.theae* in its narrower conidia 4.2–6.9 μm vs. 6.6–8.3 μm and shorter basal appendages 2–5.5 μm vs. 5–9 μm (Maharachchikumbura et al. 2014b).

## ﻿Discussion

The *Pestalotiopsis* was circumscribed by Steyaert in 1949, in honour of the Italian botanist Fortunato Pestalozza. Phylogenetic relationships within the genus and allied genera have been described, based on multigene loci (primarily ribosomal DNA sequences) and morphological characteristics. A sexual state of *Pestalotiopsis*, i.e. *Pestalosphaeria*, has been described, with the type species *P.concentrica*, originally isolated from the grey-brown spots on *Rhododendronmaximum* (Maharachchikumbura et al. 2014b). Morphological characteristics of the *Pestalotiopsis* asexual morph are primarily characterised by fusiform conidia and three pigmented median cells, each consisting of a hyaline basal cell and a hyaline apical cell with one or more simple or branched appendages, although species within this genus exhibit conidial morphological diversity and more in-depth phylogenetic analyses of different genetic loci have established that *Pestalotiopsis* comprises three distinct lineages (Jeewon et al. 2003; Maharachchikumbura et al. 2011, 2012). Based on these findings, *Pestalotiopsis* has been further divided into *Neopestalotiopsis*, *Pestalotiopsis* and *Pseudopestalotiopsis*, albeit all species within these divisions containing only four-celled conidial forms. Although mainly considered as plant pathogens, common endophytes and/or saprophytes in a variety of hosts and environments (Guba 1961; Barr 1975; Nag Raj 1993; Maharachchikumbura et al. 2014b; Li et al. 2024; Zhao et al. 2024), some *Pestalotiopsis* species can apparently cause human and/or animal diseases. These include *Pestalotiopsis* spp. isolated from bronchial samples, corneal abrasions, as well as infections of eyes, feet, fingernails, scalp and sinuses (Sutton 1999). *P.clavispora* is capable of causing fungal keratitis (Monden et al. 2013), although this latter species is also the causative agent for post-harvest stem end rot on avocado plants (Valencia et al. 2011). Some *Pestalotiopsis* species can grow aerobic and anaerobically on polyurethane as the sole carbon source and, hence, show promise in bioremediation (Russel et al. 2011). Other members of the genus have been shown to produce taxol (Gangadevi et al. 2008) and the anti-proliferative drug, chloropestolide A, has been isolated from *Pestalotiopsisfici* (Liu et al. 2009; Zhang et al. 2017).

Conidial morphology is one of the most widely used taxonomic characters for inter-specific delineation within *Pestalotiopsis* (Steyaert 1949; Guba 1961; Nag Raj 1993). However, there are considerable overlapping phenotypic characteristics that render it difficult to segregate morphologically equivocal taxa (Tejesvi et al. 2009). Conidial length and width have been emphasized as crucial characters for species identification (Steyaert 1949; Guba 1961; Mordue 1985), although reliance on these features for identification can be inaccurate. The development of molecular biology has greatly facilitated the identification of microorganisms and phylogenetic analyses of the nucleotide sequences of several genetic loci, for example, ITS, *tef1*, and *tub2*, is considered standard for fungi and can better facilitate distinctions within the current three related genera of this family, namely: *Neopestalotiopsis*, *Pestalotiopsis* and *Pseudopestalotiopsis*.

*Neopestalotiopsis* species have recently been identified as a group of emerging plant pathogens, causing severe diseases in economically important crops, particularly fruits including strawberry (Baggio et al. 2021), guava (Solarte et al. 2018; Shivangi et al. 2022), grape, mangosteen (Huanaluek et al. 2021), avocado (Fiorenza et al. 2022), blueberry (Santos et al. 2022), jabuticaba (Lin et al. 2022) and persimmon (Qin et al. 2023). Similarly, *Pseudopestalotiopsis* species, for example, *Ps.theae*, causes several diseases on host plants such as tea (*Camelliasinensis*) and *Aloevera* (Maharachchikumbura et al. 2014b, 2016; Ahmmed et al. 2022; Shahriar et al. 2022) and this fungus has been frequently isolated as an endophyte from hosts such as *Camellianitidissima*, *C.sinensis*, *Holarrhenaantidysenterica*, *Podocarpusmacrophyllus* and *Terminalia arjuna* and as a saprophyte on *Diospyroscrassiflora* seeds (Wei et al. 2007; Douanla-Meli and Langer 2009). These findings, combined with its broad host range, suggest the existence of numerous cryptic species within *Pseudopestalotiopsis*. Consequently, the actual diversity of this genus is likely significantly underestimated (Maharachchikumbura et al. 2016). Similarly, *Ps.daweiana* was first recorded as an endophyte from healthy leaves of an unknown leaf in Myanmar (Nozawa et al. 2018). These findings suggest that these fungi may mainly associate as endophytes or saprobes, opportunistically causing disease on sick or dying leaves (or other plant structures).

Here, we add to the diversity of this group and describe three new species, namely, *Neopestalotiopsislitseae* sp. nov., *Neopestalotiopsislongqishanensis* sp. nov. and *Pseudopestalotiopsiszhangzhouensis* sp. nov. In addition to causing disease, these fungi may serve as a rich source for bioprospecting and metabolite discovery.

## Supplementary Material

XML Treatment for Neopestalotiopsis litseae

XML Treatment for Neopestalotiopsis longqishanensis

XML Treatment for Pseudopestalotiopsis zhangzhouensis

## ﻿Additional information

### Author ORCIDs

Zhi-Ang Heng https://orcid.org/0009-0003-1224-9065

Tai-Chang Mu https://orcid.org/0000-0003-0999-4884

Nemat O. Keyhani https://orcid.org/0000-0003-2855-5158

Li-Xia Yang https://orcid.org/0009-0001-5700-5836

Ming-Hai Zheng https://orcid.org/0009-0005-9398-5156

Hua-Jun Lv https://orcid.org/0009-0000-6596-5805

Zhi-Ying Zhao https://orcid.org/0009-0009-3057-958X

Yu-Chen Mao https://orcid.org/0009-0007-6368-5982

Jun-Ya Shang https://orcid.org/0009-0007-0859-3604

Jiao Yang https://orcid.org/0009-0004-7976-4608

Hui-Li Pu https://orcid.org/0009-0009-4992-6561

Yong-Sheng Lin https://orcid.org/0009-0004-2639-7576

Meng-Jia Zhu https://orcid.org/0009-0009-0033-2332

Yu-Xiao Dang https://orcid.org/0009-0004-9577-7468

Dong-Mei Wu https://orcid.org/0009-0006-4126-0767

Zhen-Xing Qiu https://orcid.org/0009-0001-4057-5103

Jun-Zhi Qiu https://orcid.org/0000-0003-2293-2055

Xia-Yu Guan https://orcid.org/0009-0007-3847-7663

### Data availability

All of the data that support the findings of this study are available in the main text.
